# Oral contraceptives modify DNA methylation and monocyte-derived macrophage function

**DOI:** 10.1186/2042-6410-3-4

**Published:** 2012-01-27

**Authors:** Ilaria Campesi, Manuela Sanna, Angelo Zinellu, Ciriaco Carru, Laura Rubattu, Pamela Bulzomi, Giuseppe Seghieri, Giancarlo Tonolo, Mario Palermo, Giuseppe Rosano, Maria Marino, Flavia Franconi

**Affiliations:** 1 National Laboratory of Sex-Gender Medicine of the National Institute of Biostructures and Biosystems, Osilo, Italy; 2 Centre of Excellence for Biotechnology Development and Biodiversity Research, University of Sassari, Sassari, Italy; 3 Porto Conte Ricerche Srl, Tramariglio, Alghero, Sassari, Italy; 4 Department of Biomedical Sciences and Centre of Excellence for Biotechnology Development and Biodiversity Research, University of Sassari, Sassari, Italy; 5 Laboratory Department, SS Annunziata Hospital, Sassari, Italy; 6 Department of Biology, University Roma Tre, Rome, Italy; 7 Department of Internal Medicine, Spedali Riuniti, Pistoia, Italy; 8 SC Diabetologia Aziendale ASL 2 Olbia, Hospital San Giovanni di Dio, Olbia, Italy; 9 Servizio di Diagnosi e Cura di Endocrinologia, Azienda Ospedaliero-Universitaria, Sassari, Italy; 10 IRCCS San Raffaele Pisana, Rome, Italy

**Keywords:** androgenic and non-androgenic progestin, combined oral contraceptive, estrogen receptors, global DNA methylation, monocyte-derived macrophages, TNFα

## Abstract

**Background:**

Fertile women may be encouraged to use contraception during clinical trials to avoid potential drug effects on fetuses. However, hormonal contraception interferes with pharmacokinetics and pharmacodynamics and modifies internal milieus. Macrophages depend on the milieu to which they are exposed. Therefore, we assessed whether macrophage function would be affected by the use of combined oral contraceptives (OCs) and if this influence depended on the androgenic or non-androgenic properties of progestin.

**Methods:**

Healthy adult women were enrolled and stratified into two groups: women who did not use OCs (Fs) and women treated with OCs (FOCs). FOCs were further stratified as a function of androgenic (FOCA^+^) and non-androgenic (FOCA^-^) properties of progestins. Routine hematological, biochemical, inflammatory and endothelial dysfunction parameters were measured. Monocyte-derived macrophages (MDMs) were evaluated for the expression and activity of estrogen receptors and androgen receptors, and release of tumor necrosis factor α (TNFα) was measured from unstimulated and lipopolysaccharide-stimulated cells.

**Results:**

As is already known, the use of OCs changed numerous parameters: the number of lymphocytes, iron levels, total iron-binding capacity of transferrin, triglycerides, high-density lipoprotein, total cholesterol, and C-reactive protein increased, while prothrombin time and alkaline phosphatase decreased. Hormonal levels also varied: cortisol was higher in FOCs, while luteinizing hormone, follicle-stimulating hormone, and testosterone were lower in FOCs. Asymmetric dimethylarginine, an index of endothelial function, was lower in FOC than in Fs, as were cysteine and bilirubin. The androgenic properties of progestins affected the activity of OCs: in particular, white blood cell count, hemoglobin, high-density lipoprotein and calcium were higher in FOCA^- ^than in FOCA^+^, whereas percentage oxygen saturation and γ-glutamyl transpeptidase were lower in FOCA^- ^than in FOCA^+^. Importantly, FOCs had a lower global DNA methylation, indicating that OC may have epigenetic effects on gene expression. OC did not modify the expression of androgen receptor but increased estrogen receptor α expression, more considerably in FOCA^+^, and decreased estrogen receptor β, more considerably in FOCA^-^. Importantly, the activation state of estrogen receptor β in FOCs was decreased, while estrogen receptor α was not active in either Fs or FOCs. Unstimulated MDMs obtained from FOCs showed higher release of TNFα in comparison with Fs. After lipopolysaccharide stimulation, the release of TNFα was significantly higher in Fs than in FOCs.

**Conclusions:**

OC use induced many changes in hematological and plasmatic markers, modifying hormonal levels, endothelial function, inflammation index and some redox state parameters, producing a perturbation of the internal milieu that impacted macrophagic function. In fact, different levels of estrogen receptor expression and release of TNFα were observed in macrophages derived from OC users. Some of the above activities were linked to the androgenic properties of progestin. Even though it is not known whether these effects are reversible, the results indicate that to avoid potential skewing of results only a single type of OC should be used during a single clinical trial.

## Background

The US Food and Drug Administration encourages the enrolment of women in clinical trials that test the efficacy and safety of pharmacological treatments [1,2]. The protocol designs emphasize the need for contraception for women of childbearing potential who participate in drug trials. Certain aspects of the contraceptive requirements for such studies do not appear to have been sufficiently considered, including the fact that hormonal contraception may interfere with pharmacokinetics or even pharmacodynamics [3]. In this context, it is important to remember that sexual hormone receptors behave as transcription factors [4] and that oral contraceptives (OCs) change the endogenous milieu by varying the activity of the pituitary-ovarian [5] and hypothalamus-pituitary-adrenal axes [6]. In addition, OCs can induce subclinical abnormalities in carbohydrate metabolism [7,8], can modify lipid metabolism [9], and are associated with elevation of C-reactive protein [10]. OCs decrease symmetric methylarginine and asymmetric dimethylarginine [11], the latter being an inhibitor of nitric oxide and an index of endothelial dysfunction [12]. OC-induced variations may increase the risk of venous thromboembolism [13,14] and elevate the prevalence of atherosclerosis and its complications in young, apparently healthy women [15,16].

Macrophages play crucial roles in atherosclerosis and immunity [17,18] and are uniquely dependent on the milieu to which they are exposed [19], which, as already mentioned, can in turn be modified by OCs [5,6,9-11,13,14]. Importantly, monocyte-derived macrophages (MDMs) express estrogen and androgen receptors [20].

Therefore, we assumed that the variation of internal milieu induced by OCs may affect the function of macrophages. For this reason we studied the influence of OCs on MDM function including the expression and the activity of estrogen and androgen receptors, together with the typical macrophage function of release of tumor necrosis factor α (TNFα) and total DNA methylation in blood cells. We selected combined OCs, which are the most commonly used birth control methods across the world [21], and also considered the androgenic or non-androgenic properties of progestin [22].

## Results

### Evaluation of the effect of OCs on routine hematological and biochemical tests

Women who had not used OCs for at least 3 months to ensure a sufficient washout period (Fs) and women treated with OCs for at least 3 months (FOCs) were matched for weight, body mass index and age (Table 1). As previously reported [10,23-26], FOCs had higher number of lymphocytes and higher levels of iron, total iron-binding capacity of transferrin, triglycerides, high-density lipoprotein, total cholesterol, and C-reactive protein than non-users, whereas prothrombin time and alkaline phosphatase were less than in Fs (data not shown).

**Table 1:** Main characteristics of each subgroup of females

Characteristic	Fs (n = 85)	FOCs (n = 77)	*P *value	FOCA^+ ^(n = 33)	FOCA ^to ^(n = 44)	*P *value
Age (years)	27.0 (18.0 to 40.0)	27.0 (20.0 to 39.0)	NS	28.0 (20.0 to 39.0)	26.0 (20.0 to 39.0)	NS
Weight (kg)	53.0 (38.0 to 78.0)	54.0 (43.0 to 75.0)	NS	54.0 (45.0 to 70.0)	55.0 (43.0 to 75.0)	NS
Body mass index (kg/cm^2^)	20.2 (17.0 to 28.0)	21.0 (17.0 to 28.0)	NS	21.5 ± 2.1	21.1 ± 2.4	NS
Hemoglobin (g/dl)	12.7 ± 1.1	12.6 ± 1.0	NS	12.4 ± 1.0	12.8 ± 0.9	0.044
Saturation (%)	24.8 ± 11.5, n = 63	22.8 ± 10.2, n = 62	NS	25.1 (3.7 to 44.5)	16.0 (5.5 to 51.9)	0.005
White blood cells (10^9 ^cells/l)	6.5 (3.62 to 11.7)	6.8 (4.1 to 12.9)	NS	6.4 ± 1.6	7.1 ± 1.4	0.031
High-density lipoprotein (mg/dl)	60.9 ± 11.2	69.3 ± 13.5	< 0.001	65.0 ± 11.6	72.6 ± 14.1	0.014
Low-density lipoprotein (mg/dl)	108.5 (68.0 to 166.0)	111.0 (35.0 to 229.0)	NS	114.5 (70.0 to 229.0)	108.5 (35.0 to 193.0)	NS
High-density/low-density lipoprotein	0.6 (0.3 to 1.3)	0.6 (0.2 to 2.0)	NS	0.6 (0.2 to 0.9)	0.6 (0.3 to 2.0)	NS
Triglycerides (mg/dl)	68.0 (33.0 to 205.0)	90.5 (42.0 to 236.0)	< 0.001	83.0 (42.0 to 174.0)	99.5 (48.0 to 236.0)	NS
Glycaemia (mg/dl)	76.0 (30.0 to 110.0)	77.0 (50.0 to 93.0)	NS	73.7 ± 9.4	77.6 ± 8.3	NS
Creatinine (mg/dl)	0.7 (0.6 to 0.9)	0.7 (0.6 to 1.0)	NS	0.7 (0.6 to 1.0)	0.71 (0.62 to 0.87)	NS
Creatinine clearance (ml/min)	101.9 ± 16.3	102.3 ± 14.6	NS	99.92 ± 14.23	104.08 ± 14.88	NS
Uric acid (mg/dl)	3.6 ± 0.9	3.4 ± 0.8	NS	3.3 ± 0.9	3.4 ± 0.8	NS
Urea (mg/dl)	27.5 (14.8 to 55.1)	28.4 (18.4 to 47.2)	NS	29.7 ± 7.8	28.8 ± 6.1	NS
Total bilirubin (mg/dl)	0.5 (0.2 to 2.1)	0.4 (0.10 to 1.9)	< 0.001	0.5 (0.1 to 0.9)	0.4 (0.2 to 1.93)	NS
Alkaline phosphatase (U/l)	59.0 (38.0 to 115.0)	55.0 (34.0 to 99.0)	0.029	53.4 ± 10.1	56.9 ± 14.6	NS
Aspartate aminotransferase (U/l)	19.0 (12.0 to 39.0)	18.0 (10.0 to 60.0)	NS	18.0 (11.0 to 29.0)	18.0 (10.0 to 60.0)	NS
Alanine aminotransferase (U/l)	17.0 (6.0 to 64.0)	15.0 (6.0 to 38.0)	NS	15.0 (6.0 to 38.0)	15.0 (7.0 to 31.0)	NS
γ-Glutamyl transpeptidase (U/l)	15.0 (7.0 to 98.0)	14.0 (7.0 to 34.0)	0.018	15.0 (7.0 to 30.0)	12.0 (7.0 to 34.0)	0.024
Calcium (mg/dl)	9.4 (8.3 to 10.4) (80)	9.4 (8.4 to 10.1)	NS	9.2 ± 0.4	9.5 ± 0.4	0.003
Sodium (mEq/l)	140.0 (134.0 to 146.0)	140.0 (135.0 to 147.0)	NS	140.0 (135.0 to 147.0)	140.0 (136.0 to 144.0)	NS
Potassium (mEq/l)	4.1 (3.5 to 4.8)	4.10 (3.5 to 6.9)	NS	4.10 (3.6 to 6.9)	4.1 (3.5 to 4.8)	NS
Prothrombin time (s)	10.7 (9.8 to 11.3), n = 71	10.4 (9.6 to 11.6), n = 68	< 0.001	10.6 ± 0.3, n = 28	10.4 ± 0.4, n = 40	NS

Data are expressed as the mean ± SD or as medians and ranges; n indicates the number of subjects used to calculate the statistics, and these may vary due to the unavailability of enough plasma to complete the analyses.

NS = not significant.

When FOCs were stratified as a function of the androgenic (FOCA^+^) and non-androgenic (FOCA^-^) properties of the progestins, we observed that white blood cell count, hemoglobin, high-density lipoprotein and calcium were higher in FOCA^- ^than in FOCA^+^, whereas percentage saturation and γ-glutamyl transpeptidase were lower in FOCA^- ^than in FOCA^+^, indicating that these parameters are influenced by the androgenic properties of progestin. Finally, triglycerides tended to be higher in FOCA^- ^than in FOCA^+ ^(Table 1).

Red blood cell count, hematocrit, mean corpuscular volume, ferritin, and the numbers of neutrophils, monocytes, eosinophils, basophils and platelets did not present any significant differences between Fs and FOCs or between FOCA^- ^and FOCA^+ ^(data not shown).

### Hormonal parameters

Hormonal statuses are shown in Table 2. FOCs, as expected, had lower testosterone, luteinizing hormone, and follicle-stimulating hormone; estradiol in many cases was under the detection limit. Cortisol was significantly higher in FOCs than in Fs. Finally, thyroid-stimulating hormone was significantly higher in FOCA^- ^than in FOCA^+^, although it did not significantly differ between Fs and FOCs.

**Table 2:** Hormonal status of each subgroup of females

Hormone	Fs	FOCs	*P *value	FOCA^+^	FOCA^-^	*P *value
Cortisol (ng/ml)	202.5 (75.5 to 373.1), n = 44	334.2 (177.0 to 584.9), n = 32	< 0.001	318.0 ± 76.6, n = 16	349.4 ± 98.2, n = 16	NS
Estradiol (pg/ml)	27.7 (6.6 to 172.0), n = 42	19.7 (5.5 to 77.0)^a^, n = 9	NS	35.1 ± 30.9, n = 4	25.9 ± 20.7, n = 5	NS
Testosterone (ng/dl)	49.9 ± 19.2, n = 33	33.9 ± 14.9, n = 27	< 0.001	31.4 ± 7.3, n = 13	36.2 ± 19.3, n = 14	NS
Luteinizing hormone (mIU/ml)	6.7 (2.6 to 16.5), n = 44	3.7 (0.2 to 10.4), n = 32	< 0.001	2.2 (0.2 to 10.4), n = 16	4.4 (0.2 to 9.6), n = 16	NS
Follicle-stimulating hormone (mIU/ml)	6.8 (2.8 to 10.7), n = 44	5.6 (1.2 to 20.2), n = 32	0.001	4.9 (1.5 to 8.5), n = 16	4.3 (1.2 to 20.2), n = 16	NS
Thyroid-stimulating hormone (μIU/ml)	1.7 (0.7 to 4.7), n = 79	1.9 (0.7 to 10.9), n = 76	NS	1.4 (0.7 to 10.9), n = 32	2.0 (0.8 to 6.3), n = 44	0.030

Data are expressed as the mean ± SD or as medians and ranges; n indicates the number of subjects used to calculate the statistics, and these may vary due to the unavailability of enough serum to complete the analyses.

^a^In many samples of oral contraceptive users, estradiol was below the detection limit (5 pg/ml), so they were excluded.

FOCA^+^/FOCA^- ^= FOCs further stratified as a function of androgenic (FOCA^+^) and non-androgenic (FOCA^-^) properties of progestins; FOCs = women treated with OCs for at least 3 months; Fs = women who had not used OCs for at least 3 months to ensure a sufficient washout period; NS = not significant; OC = oral contraceptive.

### Endothelial function

Asymmetric dimethylarginine and arginine were lower in FOCs than in Fs, whereas the asymmetric dimethylarginine/arginine ratio was higher in FOCs than in Fs. Finally, symmetric dimethylarginine was similar between Fs and FOCs. Consequently, the asymmetric dimethylarginine/symmetric dimethylarginine ratio was decreased in FOCs, whereas the asymmetric dimethylarginine/arginine ratio was increased in FOCs (Table 3). Importantly, these variations were independent of the androgenic properties of progestin.

**Table 3:** Plasma arginine and plasma methylated arginine

Arginine type	Fs, n = 72	FOCs, n = 67	*P *value	FOCA^+^, n = 28	FOCA^-^, n = 39	*P *value
Arginine (μM)	77.4 ± 15.5	55.7 ± 13.9	< 0.001	55.97 ± 13.86	55.52 ± 14.04	NS
Asymmetric dimethylarginine (μM)	0.5 (0.3 to 0.8)	0.4 (0.3 to 0.7)	< 0.001	0.44 ± 0.13	0.41 ± 0.11	NS
Symmetric dimethylarginine (μM)	0.4 (0.2 to 0.7)	0.4 (0.2 to 0.6)	NS	0.43 ± 0.09	0.40 ± 0.10	NS
Asymmetric dimethylarginine/asymmetric dimethylarginine	1.2 (0.7 to 1.9)	1.0 (0.6 to 2.4)	< 0.001	1.0 (0.6 to 2.0)	0.9 (0.6 to 2.4)	NS
Asymmetric dimethylarginine/arginine	0.006 (0.004 to 0.01)	0.007 (0.004 to 0.01)	0.026	0.008 (0.004 to 0.01)	0.007 (0.004 to 0.01)	NS

Data are expressed as the mean ± SD or as medians and ranges; n indicates the number of samples used to calculate the statistics.

FOCA^+^/FOCA^- ^= FOCs further stratified as a function of androgenic (FOCA^+^) and non-androgenic (FOCA^-^) properties of progestins; FOCs = women treated with OCs for at least 3 months; Fs = women who had not used OCs for at least 3 months to ensure a sufficient washout period; NS = not significant; OC = oral contraceptive.

### Oxidative and inflammatory parameters

Because variations in redox state have been implicated in many diseases, we investigated oxidative stress parameters. Malonyldialdehyde, an index of lipid peroxidation, was not significantly different among the groups (Table 4), whereas total bilirubin was lower in FOCs than in Fs (Table 1). Among plasma thiols, cysteine was significantly lower in FOCs than in Fs, while homocysteine, glutathione, cysteinglycine, glucylcysteine and taurine, an endogenous inhibitor of hypochlorous acid [27], were not different (Table 4). Moreover, none of the above parameters was influenced by the androgenic properties of progestin (Table 4).

**Table 4:** Plasma lipid peroxidation, thiols and taurine

Type	Fs, n = 72	FOCs, n = 67	*P *value	FOCA^+^, n = 28	FOCA^-^, n = 39	*P *value
Malonyldialdehyde (μM)	4.2 ± 1.9, n = 69	4.1 ± 1.7, n = 66	NS	3.7 (1.3 to 8.6), n = 27	3.9 (0.9 to 8.6), n = 39	NS
Homocysteine (μM)	8.7 (4.0 to 41.1)	8.3 (4.3 to 16.7)	NS	8.3 ± 2.9	8.9 ± 2.9	NS
Cysteine (μM)	195.6 (118.9 to 350.7)	176.6 (115.8 to 318.4)	0.003	181.5 ± 39.8	186.6 ± 45.4	NS
Cysteinglycine (μM)	18.7 (7.6 to 33.6)	19.5 (7.4 to 38.8)	NS	20.6 (11.4 to 38.8)	18.9 (7.4 to 33.0)	NS
Glucylcysteine (μM)	4.5 ± 1.0	4.5 ± 0.9	NS	4.4 ± 1.0	4.5 ± 0.9	NS
Glutathione (μM)	7.4 (2.7 to 14.8)	8.0 (3.9 to 14.9)	NS	8.0 ± 2.4	8.2 ± 2.0	NS
Taurine (μM)	64.1 (31.7 to 202.7)	59.6 (27.3 to 158.9)	NS	60.9 (27.3 to 146.8)	59.4 (36.3 to 158.9)	NS

Data are expressed as the mean ± SD or as medians and ranges; n indicates the number of samples used to calculate the statistics.

FOCA^+^/FOCA^- ^= FOCs further stratified as a function of androgenic (FOCA^+^) and non-androgenic (FOCA^-^) properties of progestins; FOCs = women treated with OCs for at least 3 months; Fs = women who had not used OCs for at least 3 months to ensure a sufficient washout period; NS = not significant; OC = oral contraceptive.

### Global DNA methylation

**Figure 1:**
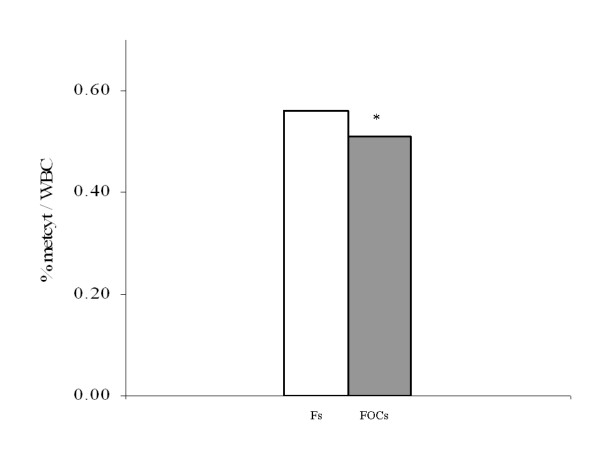
**Global DNA methylation in the two female populations**. Data are expressed as the medians of 72 Fs (white bar) and 67 FOCs (grey bar); *P = 0.033. FOCs = women treated with OCs for at least 3 months; Fs = women who had not used OCs for at least 3 months to ensure a sufficient washout period; OC = oral contraceptive.

Global DNA methylation was measured in white blood cells and expressed as percentage methylcytosine normalized to white blood cell number. Methylation was significantly higher in FOCs than in Fs (Figure 1), but the androgenic properties of progestin did not affect this parameter (data not shown).

### Expression and activities of estrogen and androgen receptors in MDMs obtained from different female populations

**Figure 2:**
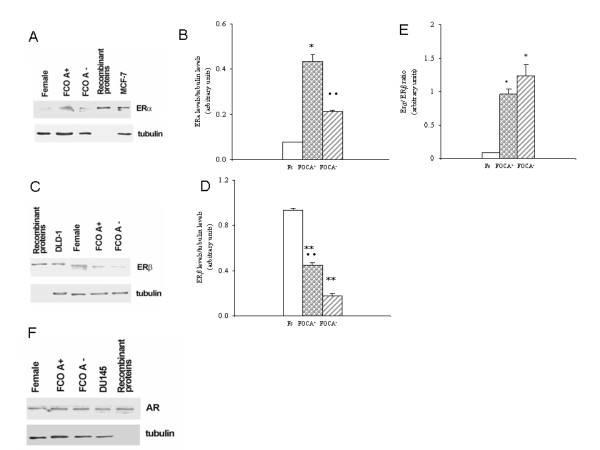
**Estrogen receptors and androgen receptor expression**. Representative western blots and corresponding densitometric analyses of estrogen receptor α **(A, B) **and estrogen receptor β levels **(C, D)**, estrogen receptor α/estrogen receptor β ratio **(E)**, and androgen receptor levels **(F)**. Panel A includes MCF-7 cells, which express estrogen receptor α, as positive control and monocyte-derived macrophages (MDMs). (B) Includes DLD-1 cells, which express estrogen receptor β, as positive control and MDMs from Fs (white bar), FOCA^+ ^(circle bar) and FOCA^- ^(striped bar). (F) Includes DU145 cells, which express androgen receptors, as positive control and MDMs. Data are expressed as the mean ± SD of at least four independent experiments. *P < 0.05; **P < 0.001 vs Fs; ^·^*P *< 0.05 between FOCA^+ ^and FOCA^-^; ^··^*P *< 0.001 between FOCA^+ ^and FOCA^-^. AR = androgen receptor; ERα = estrogen receptor α; ERβ = estrogen receptor β; ERα/ERβ = estrogen receptor α/estrogen receptor β ratio; FOCA^+^/FOCA^- ^= FOCs further stratified as a function of androgenic (FOCA^+^) and non-androgenic (FOCA^-^) properties of progestins; FOCs = women treated with OCs for at least 3 months; Fs = women who had not used OCs for at least 3 months to ensure a sufficient washout period; OC = oral contraceptive.

**Figure 3:**
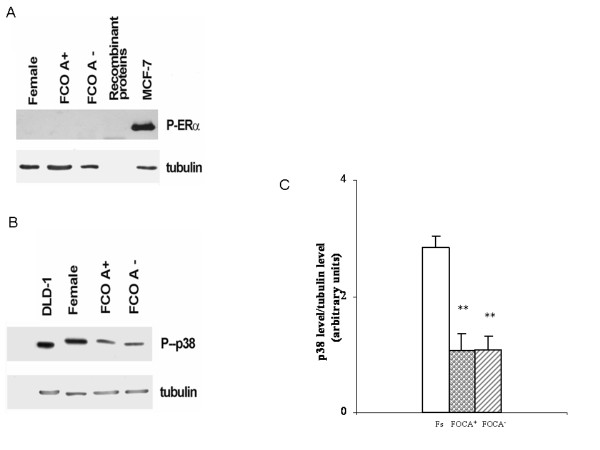
**Activation status of estrogen receptors**. Representative western blot for estrogen receptor α phosphorylation **(A) **and representative western blot with corresponding densitometric analysis of estrogen receptor β activity (measured as p38 phosphorylation) **(B, C) **in estrogen receptor α-expressing MCF-7 and estrogen receptor β-expressing DLD-1 cell lines stimulated with 10 nM estradiol (1 h) and in monocyte-derived macrophages (MDMs) from Fs (white bar), FOCA^+ ^(circle bar) and FOCA^- ^(striped bar). Data are expressed as the mean ± SD of at least four independent experiments. **P < 0.001 vs Fs. FOCA^+^/FOCA^- ^= FOCs further stratified as a function of androgenic (FOCA^+^) and non-androgenic (FOCA^-^) properties of progestins; FOCs = women treated with OCs for at least 3 months; Fs = women who had not used OCs for at least 3 months to ensure a sufficient washout period; OC = oral contraceptive.

MDMs expressed androgen receptors, estrogen receptor α, and estrogen receptor β (Figure 2), with the β isoform being the most highly expressed, as indicated comparing the estrogen receptor β band with the band obtained by loading 5 ng of recombinant proteins. Consequently, MDMs presented a low estrogen receptor α/estrogen receptor β ratio. The use of OCs had a considerable impact on estrogen receptor levels: they increased estrogen receptor α approximately fivefold and decreased estrogen receptor β approximately 0.5-fold in comparison with Fs. This effect led to a significant increase in the estrogen receptor α/estrogen receptor β ratio in MDMs derived from FOCs compared to Fs (Figure 2). When the FOCs were stratified according to the androgenic and non-androgenic properties of progestin, we observed that estrogen receptor α and estrogen receptor β levels were at least twice as high in FOCA^+ ^than in FOCA^-^. Consequently, the α/β ratio was significantly higher in FOCA^- ^than in FOCA^+ ^(Figure 2). The result obtained in FOCs prompted us to investigate whether the altered levels of estrogen receptor isoforms were paralleled by differences in their activation statuses by measuring the phosphorylation of estrogen receptor α Ser118 and the activation of p38 as a measure of estrogen receptor β activity [28,29]. The phosphorylated form of estrogen receptor α was undetectable in our samples, indicating that this receptor was inactive, whereas estrogen receptor α phosphorylation was evident in MCF-7 cells (used as a positive control). The phosphorylation of p38, and thus estrogen receptor β activation, was decreased in MDMs obtained from FOCs compared to Fs, indicating that OCs not only decreased the level but also the activity of estrogen receptor β (Figure 3). Finally, the levels of androgen receptors were not changed by the use of OCs, indicating that OCs have a specific impact on estrogen receptor levels and activities (Figure 2).

### Basal and lipopolysaccharide-induced release of TNFα from MDMs obtained from different female populations

Estrogens influence the inflammatory response through several mechanisms, including cytokine suppression [30]. This prompted us to evaluate if modifications in serum estrogen levels and in estrogen receptor expression were associated with changes in basal and lipopolysaccharide-induced release of TNFα. Despite the great variability in the basal release of TNFα, we found that basal cytokine release was consistently significantly higher in FOCs than in the Fs (Table 5). The release of TNFα was increased by lipopolysaccharide in all groups and was significantly higher in Fs than in FOCs. The androgenic properties of progestin did not influence release of TNFα.

**Table 5:** Release of tumor necrosis factor α in monocyte-derived macrophages (MDMs) obtained from each subgroup of females

Subgroup	Unstimulated (ng/ml)	Lipopolysaccharide (% increase)	*P *value
Fs, n = 76	67.4 (12.1 to 971.8)	2786.7 (323.6 to 29272.3)	< 0.001^a^
FOCs, n = 68	93.8(14.7 to 702.5)	1694.3(476.6 to 15386.7)	< 0.001^a^; < 0.02*
FOCA^+^, n = 33	90.5 (14.7 to 693.7)	1597.9 (507.3 to 10314.1)	< 0.001^a^
FOCA^-^, n = 35	88.6 (15.8 to 702.5)	1977.8 (476.6 to 15386.7)	< 0.001^a^

Data are medians and ranges; n indicates the number of samples used to calculate the statistics.

**^a^**Indicates the *P *value between unstimulated and stimulated MDMs.

*P value between FOCs and Fs.

FOCA^+^/FOCA^- ^= FOCs further stratified as a function of androgenic (FOCA^+^) and non-androgenic (FOCA^-^) properties of progestins; FOCs = women treated with OCs for at least 3 months; Fs = women who had not used OCs for at least 3 months to ensure a sufficient washout period; OC = oral contraceptive.

## Discussion

OCs modify the pituitary-ovarian axis (decreased luteinizing hormone, follicle-stimulating hormone, testosterone and estradiol), which is characteristic of the inhibition of ovulation [31,32]. The pituitary-adrenal axis (increased cortisol) is also altered by OCs and this results is in line with previous findings [6,33].

Thyroid-stimulating hormone levels were not significantly different between Fs and FOCs. However, when FOCA^- ^and FOCA^+ ^were considered, thyroid-stimulating hormone was elevated in FOCA^-^. Our results are in line with those observed by Wiegratz *et al. *, who reported that thyroid-stimulating hormone was significantly increased with the use of OCs containing a non-androgenic progestin [34].

We also confirmed that OCs induced variations in hematological and biochemical parameters, such as lymphocyte count, prothrombin time, total iron binding capacity of transferrin, C-reactive protein, and lipids [10,23-25]. Some parameters were influenced by the androgenic properties of progestin: high-density lipoproteins were higher in FOCA^-^, in accord with the findings of van Rooijen *et al. *[26], while hemoglobin, white blood cell count, calcium, percentage saturation and γ-glutamyl transpeptidase were lower in FOCA^-^, as previously reported [35].

The primary novelty of our study is the fact that OCs modified estrogen receptor α and estrogen receptor β levels, and estrogen receptor β activity, while leaving androgen receptor expression unchanged. In particular, estrogen receptor α was markedly increased, whereas estrogen receptor β was largely decreased; consequently, the ratio of α/β was greatly altered. The variations in estrogen receptor levels were associated with changes in the activation status only of estrogen receptor β. In fact, estrogen receptor α activity was undetectable in all groups, indicating that this receptor is not active in basal conditions. Conversely, p38 phosphorylation, an important step in estrogen receptor β signal transduction [4], was significantly lower in FOCs than in Fs. Importantly, the androgenic and non-androgenic properties of progestin affected only the expression of the β isoform.

OC-induced modification of hormonal levels and the estrogen receptor α/estrogen receptor β ratio was accompanied by a significant increase in basal release of TNFα. When the ratio between the two estrogen receptors was the highest, we observed the greatest release of TNFα. Interestingly, these data strongly suggest that MDMs retain a selective 'memory' of their *in vivo *environment. They also suggest that FOCs who also had high C-reactive protein levels are more prone to inflammation. In this context, it is important to remember that FOCs had higher cortisol levels, which could impact release of TNFα. It should be noted that lipopolysaccharide-induced release of TNFα was higher in Fs and that Fs had lower cortisol and higher estradiol. The influence of sex hormones on release of TNFα has been suggested by Amory *et al. *[36], and recently a direct correlation between estrogen receptor α expression and the suppression of lipopolysaccharide-induced CXCL8 secretion has been shown [20], but because estrogen receptor α was not active in our samples, we believe that the cortisol increase is of some importance. These results are in line with the OC-induced modifications of human T lymphocytes [37]. Notably, the release of cytokines by macrophages and monocytes appears to be an endocrine phenomenon. Generally, when estrogen is elevated, resting peripheral blood monocytes release less interleukin-1β and TNFα [38,39]. Moreover, monocytes obtained from surgically postmenopausal women release a higher amount of cytokines, and importantly, the administration of estrogen restores premenopausal cytokine levels [40]. When peripheral blood mononuclear cells are stimulated with lipopolysaccharide, mRNA expression and secretion of interleukin-1β and TNFα are increased in the luteal phase compared with the follicular phase [41].

Another important result of this study is the fact that OCs ameliorate endothelial function, as indicated by decreased asymmetric dimethylarginine. This marker of endothelial function is also an independent predictor of cardiovascular events and mortality [12]. Although the absolute reduction of this endogenous inhibitor of nitric oxide synthase was small, the biological variation in the plasma asymmetric dimethylarginine/arginine ratio is also very low and even a slight increase in the asymmetric dimethylarginine/arginine ratio is associated with an elevated risk of acute coronary events [12]. The reduction in the asymmetric dimethylarginine/arginine ratio is in line with the results of Valtonen *et al. *[11]. Notably, the ratio increase was mainly sustained by a decrease in arginine. The decrease in arginine is not a universal finding [11], and the reasons for this discrepancy are not well understood, but a decrease in arginine has been reported after oral hormonal replacement therapy [42].

The reduction in total DNA methylation in FOCs was small but significant. Variations in DNA methylation imply heritable epigenetic changes in gene function [42]. Notably, global hypomethylation predisposes to age-related chronic diseases, including atherosclerosis [43,44]. The global hypomethylation of DNA and reduction of asymmetric dimethylarginine occurred in the presence of a significant variation of homocysteine; previously it has been shown that folate does not differ between OC users and non-users [45], suggesting that the decreases in asymmetric dimethylarginine and DNA methylation are not attributable to a decrease in folate. In our opinion, the low levels of cysteine and DNA methylation suggest a slowdown during the demethylation and trans-sulfuration phases of the methionine cycle, which can reasonably produce a decrease in asymmetric dimethylarginine, although it is not possible to exclude other mechanisms.

When the study population was stratified for OC use, the results obtained were mainly in line with results in the literature, indicating that the sample number was sufficient to discriminate differences. Indeed, the further stratification into FOCA^+ ^and FOCA^- ^groups might have influenced the statistical power, and therefore, further differences due to the activity of progestin may not have been detected. Some information was self-reported data, which contains several potential sources of bias. Another caveat is the lack of randomization and the fact that the study enrolled women who were treated with several OCs that contained different progestin-based molecules with androgenic and non-androgenic properties. However, the current study specifically focused on evaluating factors related to MDM function in a real population and whether function was affected by OC treatment, as well as investigating whether OCs could also produce alterations in cell functions, thereby affecting the pharmacodynamics of the drugs under examination.

## Conclusions

In summary, these findings suggest that contraceptive therapy impacts the function of cells (MDMs) that play crucial roles in immunity and atherosclerosis [17,18], modifying the release of TNFα. Notably, the androgenic properties of progestin alter several properties of MDMs. OCs modify total DNA methylation, and this epigenetic change occurs independently of the androgenic properties of progestin. Considering that the preparation of MDMs from monocytes requires 10 days, it is also conceivable that the modifications induced by OCs are long lasting. At the moment, it is not known whether these effects are reversible.

Intuitively, these modifications could have a relevant impact on pharmacological targets. The impact may be different according to the androgenic properties of progestin. This implies that a single type of OC should be used during a single trial, because the type of OC used could influence the results of the designed study. We are also confident that bringing attention to this problem could help to improve drug therapy in women, who currently experience almost twice the number of adverse events as men [3].

## Methods

### Ethics statement

The local ethical committee of Azienda Ospedaliero-Universitaria of Sassari approved this study. Informed consent taking and blood sampling were performed at a voluntary blood donation session. Women willing to donate blood were asked to donate during the follicular phase of their menstrual cycle and informed that an aliquot of blood would be kept for the study. Blood chemistry tests were performed as a scheduled service for blood donors.

### Subjects

A total of 162 healthy adult women (85 non-users of OCs and 77 OC users) with regular menstrual cycles (28 days) aged 27 years (range 18 to 39) were enrolled during the period July 2007 to November 2010 at the Servizio di Diagnosi e Cura di Endocrinologia, Azienda Ospedaliero-Universitaria, Sassari. Weight and height were used to calculate body mass index. Women were defined as healthy after a physical examination and after blood chemistry analysis.

Women were free of kidney, liver, heart, and endocrine diseases and infective diseases for at least 2 months prior to the study and did not use chronic pharmaceutical treatments, with the exception of OCs. The population was stratified into two groups: Fs and FOCs, as defined earlier. This was due to the fact some women may need to change the type of OC in use to optimize the anticonceptional therapy. FOCs were further stratified as a function of the androgenic (FOCA^+^) and non-androgenic (FOCA^-^) properties of the progestins. OCs belonged to the new generation and contained ethinylestradiol, for which the most representative dose was 20 μg (n = 42). The most used progestin dose was 3 mg (n = 32); the androgenic progestins used were gestodene (the most frequently used), desogestrel, and levonorgestrel, whereas drospirenone and clormadinone have no androgenic properties [22], with drospirenone used most frequently.

### Biochemical and hematological examinations

Laboratory assessments of several biomarkers were conducted on 10 ml of fasting blood samples (8.00 am and 10.00 am) obtained from the antecubital vein of women in the follicular phase of their menstrual cycle (1 to 10 days). Blood was put in tubes with the appropriate anticoagulant (sodium citrate for coagulation, silicone coating for serum determinations and potassium-ethylenediaminetetra-acetic acid (EDTA) for all the other assessments). Plasma was aliquoted, stored at -80°C and used within 1 month to measure cysteine, homocysteine, glutathione, cysteinglycine, glucylcysteine, arginine, asymmetric dimethylarginine and symmetric dimethylarginine according to the method of Zinellu *et al. *[46-48] and to measure malonyldialdehyde according to the method of Esterbauer and Cheeseman [49], with slight modifications. Other plasma aliquots were immediately used for measuring fasting glucose, total cholesterol, low-density lipoproteins, high-density lipoproteins, triglycerides, creatinine, uric acid, urea, total bilirubin, aspartate aminotransferase, alanine aminotransferase, γ-glutamyl transpeptidase, alkaline phosphatase, calcium, sodium, potassium, sideremia, ferritin, C-reactive protein, prothrombin time, total iron binding capacity of transferrin and percentage saturation using standard laboratory procedures. Full blood aliquots were used to measure red cell count, leucocyte formula, platelet count, hemoglobin, hematocrit, and mean corpuscular volume. White blood cells were also used to determine the degree of global DNA methylation as previously described [50]. Aliquots of serum were also prepared to measure hormones. In particular, cortisol (Cortisol RIA CT, Chematil S.r.L., Angri, Italy), thyroid-stimulating hormone (VITROS TSH, Ortho-Clinical Diagnostics Johnson & Johnson, Roma, Italy), estradiol (Estradiol MAIA, Adaltis Italia S.p.A., Bologna, Italy) and testosterone (Testosterone RIA CT, RADIM S.p.A, Pomezia, Italy) were measured by RIA using commercial kits, while luteinizing hormone (LH IRMA kit, Immunotech a.s., Milano, Italy) and follicle-stimulating hormone (FSH IRMA kit, Immunotech a.s.) were detected by IRMA using commercial kits. Intra-assay and interassay coefficients of variation were less than 2.5%.

### Human monocyte isolation and MDM differentiation

An aliquot of blood taken from healthy volunteers (30 ml) was used for the isolation of monocytes. Briefly, blood was diluted with phosphate-buffered saline (pH 7.4), layered over a histopaque (density 1.077 g/cm^3^, Sigma Aldrich, Milano, Italy) gradient solution, centrifuged (400 *g*, 25 min, room temperature (RT)) and recovered by thin suction at the interface. The mononuclear cell layer was washed twice by centrifuging with phosphate buffer (200 *g*, 10 min, RT) and then resuspended in RPMI 1640 medium (Invitrogen, S. Giuliano Milanese, Italy) supplemented with 20% heat-inactivated fetal bovine serum (Invitrogen, S. Giuliano Milanese, Italy), 2 mM glutamine, 10 mM 4-(2-hydroxyethyl)-1-piperazine-ethanesulfonic acid (HEPES, Sigma Aldrich, Milano, Italy), and 1% antibiotic/antimycotic (Invitrogen, S. Giuliano Milanese, Italy). Purified monocytes were obtained by adhesion; non-adherent cells (mainly lymphocytes) were removed by gentle washes with phosphate buffer. MDMs were prepared from monocytes cultured for 8 to 10 days in a 5% CO_2 _incubator at 37°C in RPMI 1640 medium containing 20% fetal bovine serum, 2 mM glutamine, 10 mM HEPES and antibiotics/antimycotics; medium was changed every 2 to 3 days. MDMs were prepared and characterized as described [51].

### Expression and activation status of estrogen receptor α, estrogen receptor β and androgen receptor in MDMs derived from different female populations

An aliquot of MDMs was lysed, seeded in six-well plates, washed twice with ice-cold phosphate-buffered saline (PBS) and scraped in lysis buffer (20 mM Tris-HCl (pH 7.5), 150 mM NaCl, 1 mM Na_2_EDTA, 1 mM ethylene glycol tetra-acetic acid (EGTA), 1% Triton X, 2.5 mM sodium pyrophosphate, 1 mM β-glycerophosphate, 1 mM Na_3_VO_4_, 1 μg/ml leupeptin and 1 mM phenylmethanesulfonylfluoride (PMSF), Sigma Aldrich, Milano, Italy). The lysate was centrifuged (13,000 *g*; 10 min, 4°C); the supernatants obtained were collected and stored at -80°C until western blot analyses for estrogen receptor α, estrogen receptor β and androgen receptor [29]. To measure estrogen receptor α and estrogen receptor β, electrophoreses were performed in the presence of 5 ng of recombinant estrogen receptor α and estrogen receptor β.

The activation states of estrogen receptors were evaluated by analyzing the phosphorylation of Ser118 of estrogen receptor α and the phosphorylation of p38 for estrogen receptor β, according to [4]. Protein concentration was determined with the Pierce BCA protein assay kit (Thermo Scientific, Celbio SPA, Pero, Italy). Moreover, a standard curve of recombinant proteins showed that the band intensity was proportional to the protein quantity. Antibody reaction was visualized with chemiluminescence Western Blotting Detection Reagent (Amersham Biosciences, Little Chalfont, UK). Densitometric analyses were performed with ImageJ software for Windows http://rsbweb.nih.gov/ij/.

### Spontaneous and lipopolysaccharide-induced release of TNFα from MDMs derived from different female populations

MDMs (1.4 × 10^4^) were incubated for 24 h in the absence or presence of 100 ng/ml lipopolysaccharide (Sigma Aldrich, Milano, Italia), a component of Gram-negative bacterial cell walls, that binds to Toll-like receptor 4 [52]. Supernatants were then collected and stored at -80°C and used to measure TNFα using a commercial ELISA kit (human TNFα/TNFSF1A DuoSet ELISA kit, R&D Systems, Milano, Italy) following the manufacturer's instructions.

### Statistical analysis

Statistical analysis was performed by comparing Fs versus FOCs and FOCA^+ ^versus FOCA^-^. Continuous parametric variables were analyzed using the Student's t test. Non-parametric variables were compared between groups by the Mann-Whitney rank test. For all tests, a *P *value ≤ 0.05 was considered statistically significant.

## Competing interests

The authors have no conflict of interest that could be perceived as prejudicing the impartiality of the research reported.

## Authors' contributions

FF, GT, MM, GR and MP conceived and designed the experiments. IC, MS, AZ, LR, CC, PB, MP and MM performed the experiments. GS, CC, IC and MS analyzed the data. IC, FF and MM wrote the paper. All authors read and approved the final manuscript.
